# Ixodid Tick Vectors of Wild Mammals and Reptiles of Southern India

**Published:** 2018-09-30

**Authors:** K. G. Ajith Kumar, Reghu Ravindran, Joju Johns, George Chandy, Kavitha Rajagopal, Leena Chandrasekhar, Ajith Jacob George, Srikanta Ghosh

**Affiliations:** 1 Department of Veterinary Parasitology, College of Veterinary and Animal Sciences, Pookode, Lakkidi, Kerala, India; 2 Centre for Wildlife Studies, College of Veterinary and Animal Sciences, Pookode, Lakkidi, Kerala, India; 3 Department of Livestock Products Technology, College of Veterinary and Animal Sciences, Pookode, Lakkidi, Kerala, India; 4 Department of Veterinary Anatomy, College of Veterinary and Animal Sciences, Pookode, Lakkidi, Kerala, India; 5 Department of Veterinary Pathology, College of Veterinary and Animal Sciences, Pookode, Lakkidi, Kerala, India; 6 Entomology Laboratory, Division of Parasitology, Indian Veterinary Research Institute, Izatnagar, India

**Keywords:** Ticks, Wild mammals, Reptiles, Wayanad, South India

## Abstract

**Background:**

We aimed to focus on the ixodid ticks parasitizing wild mammals and reptiles from Wayanad Wildlife Sanctuary, Western Ghat, southern India.

**Methods:**

The taxonomic identification of ticks collected from wild mammals and reptiles was performed based on the morphology of adults.

**Results:**

We revealed eight species of ticks including, *Amblyomma integrum*, *Rhipicephalus* (*Boophilus*) *annulatus*, *Haemaphysalis* (*Kaiseriana*) *spinigera*, *H.* (*K.*) *shimoga*, *H.* (*K.*) *bispinosa*, *H.* (*Rhipistoma*) *indica*, *Rhipicephalus haemaphysaloides* and *R. sanguineus* s.l. collected from nine species of wild mammals while four tick species *Ablyomma kraneveldi*, *A. pattoni*, *A. gervaisi* and *A. javanense* parasitizing on four species of reptiles. The highest host richness was shown by *H.* (*K.*) *bispinosa* and *R. haemaphysaloides* parasitizing six and five different host species, respectively. Reports of *R.* (*B.*) *annulatus* on sambar deer, *A. javanense* and *A. kraneveldi* on python as well as *A. pattoni* on Indian rat snake are the new host records from this region.

**Conclusion:**

Eight species of ticks parasitizing on nine species of wild mammals and four species of parasitizing on four species of reptiles were identified. The highest host richness was shown by *H.* (*K*.) *bispinosa* and *R. haemaphysaloides. H. spinigera* as the vector of KFD was also identified in this study.

## Introduction

Ticks (Ixodida) are obligate, non-permanent ectoparasites of terrestrial vertebrates (1). They are exclusively haematophagous in all feeding stages of their life cycle and have considerable medical and veterinary importance (2). Besides, causes great economic losses to the livestock worldwide (3). Currently, 904 valid tick species have been listed throughout the world (4–13). Ticks parasitize a wide range of vertebrate hosts and transmit a variety of pathogenic agents than any other group of arthropods (14, 15). Heavy infestation can cause blood loss, reduced weight gain and lowered milk production, even some tick species downgrade quality of hides (16). It is estimated that 80 per cent of world’s livestock population is suffering from the deleterious effects of ticks (17).

Nearly, 106 Argasid and Ixodid tick species infesting domestic, wild and game animals were documented from India (18). The ixodid tick *R*. (*B*.) *microplus* is the most prevalent and economically important species infesting livestock in India (19). On the global basis the losses incurred by livestock industry due to TTBDs was estimated in the range of 14000 to 18000 million US $ / year (16). The annual cost of control of TTBDs in India has been estimated as US $ 498.7 million (20). From the stand point of global biodiversity conservation, ticks are playing a significant role, as they are able to affect the fitness of wild life species by spill over epizootic outbreaks (21). Moreover, wild animals can act as reservoirs of infectious organisms and ticks can transmit them into domestic animals and humans. Over the last few decades approximately 75 per cent of emerging diseases, including zoonoses, having wildlife origin (22).

Western Ghats or the Sahyadri of southern India with an area of 17,000km^2^ run parallel to the west coast of peninsular India stretching from Cape Comorin (or Kanyakumari) in the south to the Surat Dangs in Gujarat in the North. Human and livestock population existing as high densities in this region (23, 24). Wayanad Wildlife Sanctuary (76°02′ to 76° 27′ East longitude and 11° 35′ to 11° 51′ North latitude) with an area of 344 sq. km. is set lofty on the majestic Western Ghats with altitude ranging from 650 to 1150m above the sea level. Rich in wild animals biodiversity, the sanctuary is an integral part of the Nilgiri biosphere reserve.

Deadly tick borne viral infections like Kyasanur forest disease (KFD) were reported from humans in Karnataka (25) and Kerala (26–27) with or without mortality and Crimean-Congo haemorrhagic fever (CCHF) from Gujarat while CCHFV-specific antibodies were detected in human samples from Kerala (28). Previous reports on tick vectors of wildlife of southern India are scanty.

Hence, an active surveillance was initiated to document the possible ixodid tick vector species from the free ranging mammals and reptiles of the Western Ghats of Wayanad of Kerala, India.

## Materials and Methods

### Study area

Study are comprised of the entire Wayanad Wildlife Sanctuary (76° 02′ and 76° 27′ East Longitude and 11° 35′ and 11°51′ North Latitude) and adjoining area in the Wayanad District of Kerala, India.

### Animal and tick collection

Wild animals are regularly brought to the College of Veterinary and Animal Sciences, Pookode by the officials of Department of Forest, Kerala for postmortem examination (animals died due to hunterattack, malicious poisoning or trapped), treatment and for health checkup prior to release back into the forest. Dead animals are surveyed in a short-time window (within 24 hoursafter death). A total of 46 wild mammals of 16 different species and 23 reptiles of nine species were included in the present study (Table 1). Body of these animals was examined for the presence of adults and engorged nymphs of tick. Adult ticks were collected in glass tubes and immediately transported to the parasitology laboratory for identification. If identification was not possible on day of collection, the collected ticks were stored for 24h in Boardman’s solution I (17% ethanol, 3% ether and 80% water). Then, for long term storage, they were transferred to solution II (80% ethanol, 15% water, 5% glycerol) to which 1% chlorform is added to prevent the colour change. Engorged live nymphs were immediately placed in BOD incubator at 28 °C and RH 85% for moulting to adults.

**Table 1.** Species of ticks detected on the wild mammals and reptiles of Wayanad region of Western Ghats

**No.**	**Name of wild animal host examined**	**Number of host examined**	**Tick Species**	**No. of tick collected**	**Life stage (Nymph (N)/Adults(A))**
**1**	Sambar deer [*Cervus unicolor* Kerr, 1792]	11	*Amblyomma integrum* Karsch, 1879	85	Adult
			*Rhipicephalus* (*Boophilus*) *annulatus* Say, 1821	36	Adult
			*R. haemaphysaloides* Supino, 1897	40	Nymph
			*H.* (*Kaiseriana*) *bispinosa* Neumann, 1897	96	Adult
			*Haemaphysalis* (*Kaiseriana*) *spinigera* Neumann, 1897	35	Adult
			*H.* (*K*.) *shimoga* Hoogstraal and Trapido, 1964	27	Adult
				10	Adult
**2**	Spotted deer [*Axis axis* (Erxleben, 1777)]	2	*R*. (*B.*) *annulatus* Say, 1821	10	Adult
			*R. haemaphysaloides* Supino, 1897	14	Adult
			*H*.(*K*.) *bispinosa* Neumann, 1897	26	Adult
**3**	Barking deer [*Muntiacus muntjak* (Zimmermann, 1780)]	4	*R.* (*B.*) *annulatus* Say, 1821	5	Adult
			*R. haemaphysaloides* Supino, 1897	12	Adult
			*H.*(*K*.) *bispinosa* Neumann, 1897	42	Adult
**4**	Mouse deer [*Moschio-laindica* (Gray, 1852)]	2	*H.*(*K*.) *bispinosa* Neumann,	20	Adult
			1897*Haemaphysalis* (*K*.) *spinigera* Neumann, 1897	5	Adult
**5**	Gour [*Bos frontalis* Lambert, 1804]	1	*H. (K.) shimoga* Hoogstraal and Trapido, 1964	5	Adult
**6**	Wild pig [*Sus scrofa* Linnaeus, 1758]	4	*Amblyomma integrum* Karsch, 1879	10	Adult
			*R. haemaphysaloides* Supino, 1897	10	Adult
			*R. sanguineus* s.l. Latreille, 1806	5	Adult
**7**	Tiger [*Panthera tigris* (Linnaeus, 1758)]	1	*H.* (*K*.) *bispinosa* Neumann, 1897	48	Adult
**8**	Leopard [*Pantherapardus* (Linnaeus, 1758)]	3	*R. haemaphysaloides* Supino, 1897	8	Adult
			*H.*(*K*.) *bispinosa* Neumann, 1897	10	Adult
			*H.* (*Rhipistoma*) *indica* Warburton, 1910	5	Adult
**9**	Malabar giant squirrel[*Ratufa indica* (Erxleben, 1777)]	2	*H*. (*K*.) *spinigera* Neumann, 1897	5	Adult
**10**	Leopard cat [*Prionailurus bengalensis* (Kerr, 1792)]	4	Nil	Nil	Nil
**11**	Bonnet macaque [*Macaca radiata* (Geoffroy Saint-Hilaire, 1812)]	7	Nil	Nil	Nil
**12**	Slender loris [*Loris tardigradus* (Linnaeus, 1758)]	1	Nil	Nil	Nil
**13**	Small Indian civet cat [*Viverricula indica* (Geoffroy Saint-Hilaire, 1803)]	1	Nil	Nil	Nil
**14**	Common Palm civet [*Paradoxurus hermaphroditus* (Pallas, 1777)]	1	Nil	Nil	Nil
**15**	Indian Giant Flying squirrel [*Petaurista philippensis* (Elliot, 1839)]	1	Nil	Nil	Nil
**16**	Indian Grey Mangoose [*Herpestes edwardsii* (É. Geoffroy Saint-Hilaire, 1818]	1	Nil	Nil	Nil
**17**	Monitor lizard [*Varanus bengalensis bengalensis* (Linnaeus 1758)]	2	*Amblyomma gervaisi* Lucas, 1847	8	Adult
**18**	Python [*Python molurus* Linnaeus, 1758]	5	*A. gervaisi* Lucas, 1847	5	Adult
			*A. javanense* Supino, 1897	4	Adult
			*A. kraneveldi* Anastos, 1956	3	Adult
**19**	Cobra [*Naja naja* Linnaeus 1758]	1	*A. gervaisi* Lucas, 1847	2	Adult
**20**	Indian Rat snake [*Ptyas mucosa* Linnaeus 1758]	3	*A. pattoni* Neumann, 1910	2	Adult
**21**	Russel viper [*Daboia russelii* Shaw and Nodder 1797]	1	Nil	Nil	Nil
**22**	Ceylone cat snake [*Boiga ceylonensis* (Günther, 1858)]	2	Nil	Nil	Nil
**23**	Montane Trinket Snake [*Coelognathus Helena monticollaris* (Schulz, 1992)]	3	Nil	Nil	Nil
**24**	Common Vine Snake [*Ahaetulla nasuta* Lacépéde 1789]	3	Nil	Nil	Nil
**25**	Checkered keel back [*Xenochrophis piscator* Schneider 1799]	3	Nil	Nil	Nil

### Tick identification

The taxonomic identification was performed based on the morphology of adult ticks according to standard keys and monographs (29–36).

## Results

**Fig. 1.**
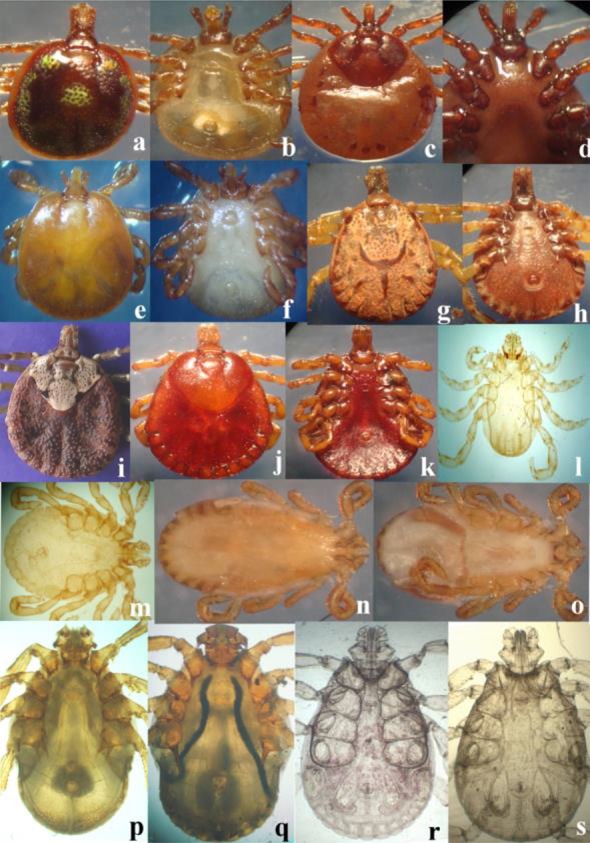
*Amblyomma gervaisi* Male: dorsal view (a) ventral view(b), *A. kraneveldi* Female: dorsal view (c) ventral view (d), *A. pattoni* Male: dorsal view (e) ventral view (f), *A. integrum* Male: dorsal view (g) ventral view (h), *A. integrum* Female: dorsal view (i), *A. javanense* Female: dorsal view (j) ventral view (k), cleared specimen of *Rhipicephalus* (*B*.) *annulatus* Male: dorsal view (l), cleared specimen of *Haemaphysalis* (*Kaiseriana*) *bispinosa* Male: ventral view (m), *H. indica-* Male: dorsal view (n) ventral view (o), *H. shimoga* Male: ventral view (p), *H.* (*Kaiseriana*) *spinigera* Male: ventral view (q): *R. haemaphysaloides* Male: ventral view (r), *R. sanguineus* s.l. Male: ventral view (s). (Figures not to scale)

Out of 46 wild mammals and 23 reptiles, 12 species of ixodid ticks belonging to five genera were identified (Table 1, Fig. 1). Of the 16 mammalian host species, seven were free from any tick infestation. Amongst the identified tick species, *Haemaphysalis* (*Kaiseriana*) *bispinosa* Neumann, 1897 was the most prevalent species while *Rhipicephalus sanguineus* s.l. Latreille, 1806 was the least. Ticks belonging to the genus *Hyalomma*, *Ixodes* and *Dermacentor* were not identified in the present study.

Amongst the tick species collected from reptiles, *Amblyomma gervaisi* Lucas, 1847 was retrieved from three species of pythons, i.e., at a time only one species of tick was collected from each python. Sambar, spotted, barking and mouse deers, wild pig, tiger and leopard were infested with more than one species of tick. A minimum of three species of ticks were retrieved from each of the 11 examined sambar deers, with a total of six species identified in them. All the wild pigs examined were parasitized by *A. integrum* and *R. haemaphysaloides* with the exception *R. sanguineus* s.l. found only in one animal. In all mammalian species, ticks were present throughout the body with more infestation on the external surface of the ear pinna and neck.

In snakes, ticks were attached between and below the scales with no ticks were seen attached to the ventral aspect of the body. Only *A. gervaisi* Lucas, 1847 could be collected from monitor lizards. Male *A. gervaisi* was collected from the lateral side of the body, axilla of the left forelimb and the periphery of cloaca / ventral depression just behind cloaca. Female ticks were collected from the axillary region and between the toes of forelimbs.

## Discussion

Among the reported species of ticks from India, *A. testudinarium*, *D. auratus*, *H. bispinosa*, *H. spinigera*, *H. intermedia*, *Hyalomma anatolicum anatolicum*, *H. marginatum isaaci*, *H. hussaini*, *H. detritum*, *H. kumari*, *B. microplus*, *I. acutitarsus*, *I. ovatus*, *N. monstrosum*, *R. haemaphysaloides* and *R. turanicus* are the most widely distributed ticks of cattle, buffalo, sheep and goat (37). Among this species, *I. acutitarsus* and *I. ovatus* were reported mainly from eastern and north-eastern states of the country (19). *Haemaphysalis bispinosa* and *R* (*B*.) *microplus* are prevalent throughout India, while *H. spinigera* is restricted to southern states, central zones, Orissa and Meghalaya (19). A total of 23 species of ticks were reported in domestic and wild animals from the different parts of Kerala State (19, 38, 39). The species of ixodid ticks reported from Kerala include, *R.*(*B*.) *annulatus*, *R*.(*B*.) *microplus*, *R*.(*B*.) *decoloratus*, *R. sanguineus* s.l., *R. haemaphysaloides*, *R. turanicus*, *H. bispinosa*, *H. intermedia*, *H. aculeata*, *H. cuspidata*, *H. knobigera*, *H. turturis*, *H. spinigera*, *H. anatolicum*, *H. marginatum isaaci*, *H. hussaini*, *A. integrum*, *N. monstrosum*, and *N. keralensis* (38, 39).

A total of 35 species of ticks were reported from sambar deer throughout its native range and introduced habitats (40) which include 11 species from two extreme ends of India, southern (comprising Karnataka and Kerala states) and the northeastern ends (Assam). The possibility of spreading of ticks from the northeastern states to the southern state is very difficult as there is no practically animal movement practically between these states. Only five species of ticks were recorded from the Karnataka and one (*H. sambar*) from Kerala (40). In the present study, six species of ticks were recorded on sambar deer from Wayanad, Kerala, none of the specimen was conforming to the morphology of *H. sambar*. As well as, in the present study, *R.* (*B.*) *annulatus* recorded for the first time on sambar deer showing the status of a new host for this species. Among all the sambar deer examined, at least three species of ticks were observed in each animal, and the presence of *A. integrum* was a constant feature. In the present study, *R.* (*B*.) *annulatus*, *R. haemaphysaloides* and *H.* (*K.*) *bispinosa* were also recorded on both spotted deer (*Axis axis*) and barking deer as previously reported by Miranpuri (41).

The presence of *A. integrum*, *R. haemaphysaloides* and *R. sanguineus* s.l. in wild boars observed in the present study so agrees with the tick-host relationship (41). Less frequency of *R. sanguineus* s.l. in wild pig in the present study corroborates with previous report (42). *Sus scrofa* is a major host for adults of *D. auratus*, which also infests bear, rhinoceros and deer of primary and secondary forests (mostly at altitude below 400m) of India, Sri Lanka, Nepal, Bangladesh, Burma, Thailand, Vietnam, Laos, Peninsular Malaysia, and Sumatra (43). We could not record this species from any wild animals. However, an adult male *D. auratus* was recently recorded from a man trekking through the forest of Wayanad region (44)

Literature reveals *H.* (*K*.) *bispinosa* was not recorded from leopards and tigers in Western Ghats(45). However, *Haemaphysalis* sp. was reported in leopard at Nagpur, Maharashtra (46). Similarly, a distinctive small member of the *H*. (*K*.) *bispinosa* group, *H.* (*K*) *ramachandrai*, was recorded on sambar deer, barking deer, chital deer, tiger, leopard, domestic cattle, buffalo and goats from forest lowlands of the Himalayan foothills of India and Nepal. *H. bipsinosa* is a ubiquitous medically important parasite of domestic animals in India transmitting various diseases in domestic animals (19). The present finding of *R. haemaphysaloides* and *H.* (*Rhipistoma*) *indica* infestations in leopard corroborated with previous findings (36, 41).

Four species of ticks were collected from four out of the eight species of snakes examined in the present study. Based on the available reports, *A. javanense* and *A. kraneveldi* on python and *A. pattoni* on Indian rat snake are the new records. Recent survey on ticks of snakes in the north Western Ghats recorded only *A. gervaisi* on two species of snakes viz., Indian rat snake and spectacled cobra. *Amblyomma gibsoni*, *A. varanensis* and *A. gervaisi* were previously reported in monitor lizard (34, 47, 48). However, *A. gervaisi* was the only tick species observed in monitor lizards in the present study.

The major infectious organisms of ruminants transmitted by common tick species in India are, *Theileria annulata* (transmitted by *Hyalomma anatolicum* and *H. marginatum isaaci*), *Babesia bigemina*, *Anaplasma marginale* and *Ehrlichia bovis* (transmitted by *R.* (*B*.) *microplus*), *B. motasi* (transmitted by *Haemaphysalis* spp.) and *B. ovis* (transmitted by *Rhipicephalus* spp.) (37). The occurrence of *T. annulata* and *B. bigemina* was reported from the whole India while *A. marginale*, *E. bovis* and *E. phagocytophila* is confined to some restricted zones. *Hepatozoon canis*, *Ehrlichia canis*, *Mycoplasma haemocanis*, *Anaplasma platys*, *B. vogeli* and *B. gibsoni* are the TBD pathogens found infecting dogs in India due to the potential tick vectors, *Rhipicephalus* (most commonly) and/or *Haemaphysalis* ticks (49).

In humans, Lyme disease, Kyasanur Forest Disease (KFD), Crimean-Congo Hemorrhagic Fever (CCHF) and babesiosis are some of the important tick borne zoonoses reported from India (25, 50). Human babesiosis and CCHF were reported from Gujarat state (50, 51) of northern India. Kyasanur forest disease (KFD) was originally recognized as a febrile illness in the Shimoga district of Karnataka state of India (52). During 2013, only single case of Kyasanur forest disease (KFD) was reported without any mortality in humans from Wayanad, Kerala (26) while eleven confirmed cases, one death and eight suspected cases were already reported in the month of February 2015 (27–29). The principal vector for KFD, *H.* (*K*.) spinigera was identified in the present study. *Dermacentor auratus* reported previously from a human (44) from Wayanad can also act as vector for the disease. Hence, it could be possible that KFD may spread into more and more areas of Kerala in future. Lyme disease in humans was documented from northern and north eastern India (53, 54). Lyme disease was reported recently from Wayanad too (26, 55) even though its tick vector could not be established.

The information gathered in the present study will be useful for public health specialists, medical professionals, zoologists, parasitologists and other professionals for designing tick control strategies for the entire southern India to prevent the possible emergence of newer tick borne diseases especially zoonoses.

## Conclusions

Twelve species of ticks from wild mammals and reptiles were recorded from southern India suggesting the contribution of wild life for tick abundance and prevalence in the tick fauna of this region. *Haemaphysalis* (*K*.) *bispinosa* was common among the wild ungulates and the large carnivores. As well as, *H.* (*K*.) *spinigera*, the principal vector for Kyasanur Forest disease (KFD) was identified in the present study. The data presented will be helpful for designing ticks and tick-borne disease control programs in this region of the country.
